# A mobile isolator that eases the life of ICU staffs during COVID-19

**DOI:** 10.1186/s13054-022-04146-2

**Published:** 2022-09-20

**Authors:** Linghua Yu, Feng Han

**Affiliations:** Gastroenterology and Hepatology Department, Institute of Liver Diseases, The Affiliated Hospital of Jiaxing University, 1882 Central-South Road, Jiaxing, 314001 Zhejiang Province People’s Republic of China

**Keywords:** COVID-19, Mobile isolator, Intensive care unit

During the outbreak of COVID-19, more patients need to be admitted to the intensive care unit (ICU) for treatment and rescue [1]. While the introduction of COVID-19 has been considered a new threat to the safety of medical workers in the ICU, personal protective equipment (PPE) for them is particularly important to avoid nosocomial infection during a COVID epidemic [2]. In many cases, health care staffs are afraid to get in and out of ICU for the risk of being exposed to COVID-19. Thus, effective strategy and measures have to be adopted to prevent transmission of COVID between patients and health care workers in ICU.

Survey showed it is a terrible experience to wear a disposable protective clothing for long period due to heat retention and sweating. Besides, it was time-consuming to put on and take off these clothing. These weak points seriously affect the protective effect of PPE. Meanwhile, the extensive use of disposable protective clothing during the epidemic has raised concerns over mental health issues among medical workers in ICU, who are required to wear these sealed clothing every day [3].

The disposable protective clothing cannot be recycled after use because it is contaminated with virus droplets or blood splashes. They must be treated as infectious waste and treated in a high-temperature incinerator with strict specifications. The increased demand for disposable protective clothing is only a good news for manufacturers, but it poses a huge burden on the environment.

Researchers proposed the deployment of a mobile pod for containing and treating COVID-19 patients in an underserved community area as a standard method of treating highly contagious and severely ill patients during a pandemic (4). Herein, we designed a mobile transparent isolator, which can carry the medical worker going around the ICU (Fig. 1). This mobile isolator has a segway-like chassis and a transparent isolating cabin. The chassis can move forward, backward, and turn under the control of the passenger. The cabin accommodates only one medical worker, which allows it to move agilely between ward beds. So cabin can be disinfected repeatedly, and patients can be treated quickly without the need for disposable protective clothing. The mobile isolator is equipped with sensors that monitor the changes in temperature, pressure, or moisture. It will stop or reverse if necessary to avoid potential collision. When detecting an obstacle, it will automatically start rotating to ensure its safe movement. Equipped with this mobile isolator, medical workers facing highly contagious patients do not require long-term isolation in wards or hotels, which greatly alleviates mental illness. In addition, the mobile isolator possesses advantages over the PPE that it has low TCO (total cost of ownership) and low social cost. And the medical worker will not feel anxious about a possible shortage of supplies. Needless to say, this mobile isolator can also be used in any outbreak of infectious diseases in ICU, such as the recent monkeypox.

**Fig. 1:**
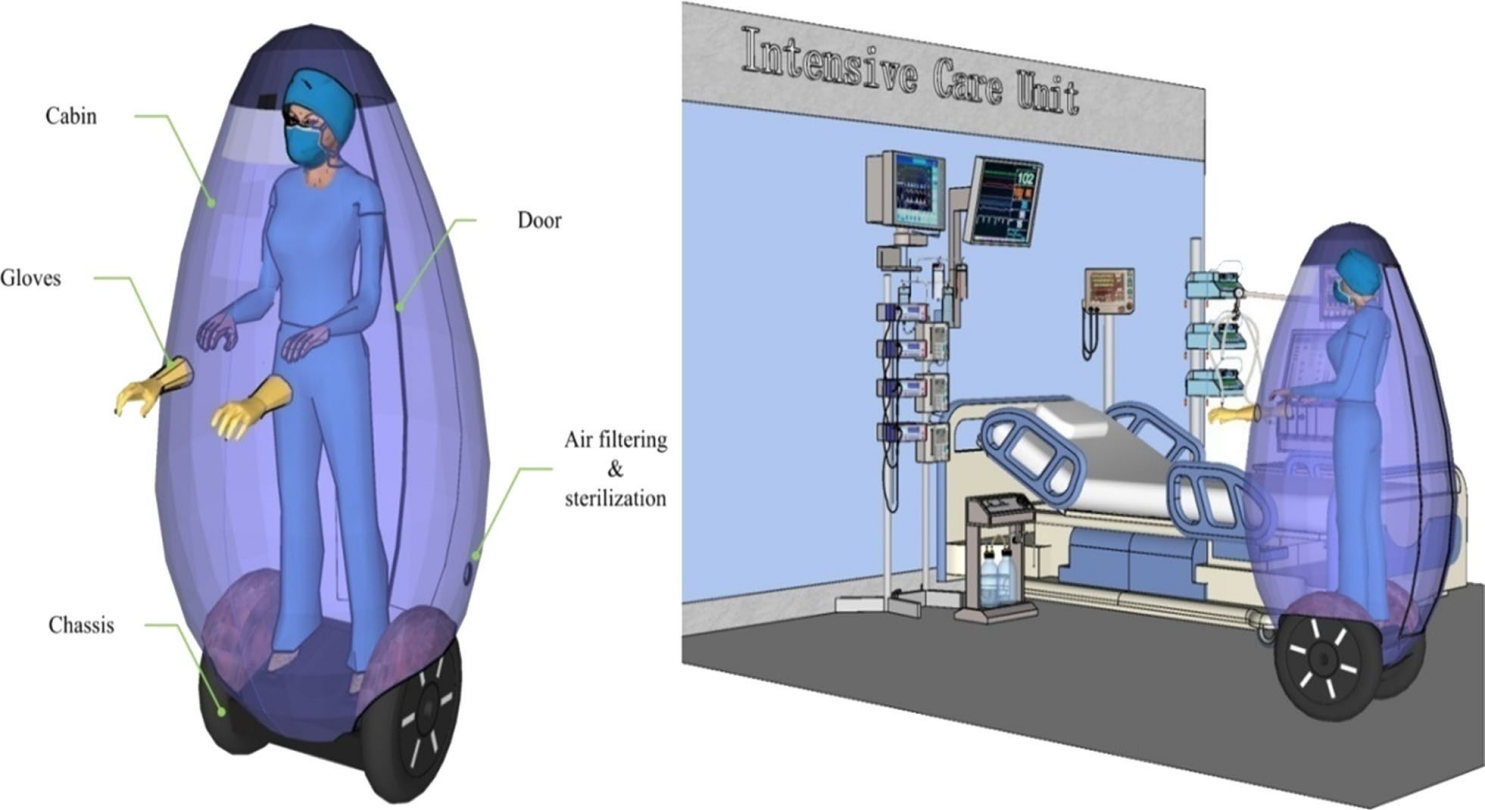
The illustration of a mobile isolator in ICU

## Data Availability

Not applicable.
